# Urinary Iodine Concentration in a Cohort of Adult Outpatients with Thyroid Diseases in Liguria 14 Years after the Law on Salt Iodization

**DOI:** 10.3390/nu12010011

**Published:** 2019-12-19

**Authors:** Lucia Conte, Martina Comina, Eleonora Monti, Marilena Sidoti, Ornella Vannozzi, Lucia Di Ciolo, Flavia Lillo, Massimo Giusti

**Affiliations:** 1 Department of Internal Medicine, University of Genoa, 16132 Genoa, Italy; luciaconte88@hotmail.it (L.C.); martina.comina@gmail.com (M.C.); 2 Endocrine Unit, San Martino Polyclinic Hospital, 16132 Genoa, Italy; eleonora.monti87@gmail.com; 3 Endocrine Ambulatory, Centro Diagnostico Priamar, 17100 Savona, Italy; marilena.sidoti@tiscali.it; 4 Laboratory Unit, San Martino Polyclinic Hospital, 16132 Genoa, Italy; mariaornella.vannozzi@hsmartino.it; 5 Nuclear Medicine Unit, Santa Corona Hospital, Pietra Ligure, 17027 Savona, Italy; l.diciolo@asl2.liguria.it; 6 Laboratory Unit, Azienda Sanitaria Locale 2 of Liguria, 17100 Savona, Italy; f.lillo@asl2.liguria.it

**Keywords:** urinary spot iodine, adult subjects, Ligurian districts, thyroid diseases

## Abstract

Italy is considered a mildly iodine-deficient country. The aim of this study was to evaluate the iodine status of a cohort of adults living in Liguria after the 2005 salt iodization program. We searched all medical records of patients examined in two endocrine outpatient clinics in Genoa and Savona for data on urinary iodine. Subjects were under evaluation for thyroid diseases. Information on the type of salt used was found in few clinical records. Iodized salt use was reported in 29%, 20%, and 13% of records of people living in Genoa districts, the Savona district and nearby districts, respectively. The average urinary iodine concentration was 112.9 ± 62.3 µg/L (*n* = 415, median 101.0 µg/L). Non-significant differences (*p* > 0.05) were found between subjects with (median 103.5 µg/L) and without (median 97.5 µg/L) a thyroid gland, between the periods 2009–2013 (median 105.0 µg/L) and 2014–2018 (median 97.5 µg/L), and between Genoa (median 94.0 µg/L), Savona (median 105.0 µg/L) and the other districts (median 114.5 µg/L). No correlation with age, body mass index, creatinine, free thyroxine, thyroglobulin, levo-thyroxine dosage, or thyroid volume was observed. These data suggest a borderline status of iodine sufficiency in this cohort.

## 1. Introduction

Iodine deficiency is an important issue for public health, since it is involved in thyroid diseases, reproductive impairment, and various degrees of cognitive disability [1,2].

To reduce iodine-deficiency disorders (IDD), in the last 25 years both governmental and non-governmental organizations have implemented iodine fortification strategies and monitoring programs. The preferred strategy for the control of IDD is currently universal salt iodization [3]. Except for rare transient iodine-induced thyrotoxicosis in severely iodine-deficient areas, no adverse effects have been observed [4,5].

As a result of iodine fortification programs implemented over the last two decades, some countries, such as Iran [6] and the Netherlands [7], have achieved the target of iodine sufficiency and IDD control. In Italy, IDD have been documented since ancient times, and a nationwide salt iodization program was implemented in 2005 after the approval of Law no. 55/2005. The Italian salt iodization program mainly concerns table salt, though salt used in industry is also regulated by law [5]. However, there is still a situation of mild iodine deficiency in the country [8]. Most data on iodine intake in Italy are collected in schoolchildren and women of reproductive age, which are the two groups most sensitive to the lack of iodine. Although these data have documented improvements in the last 5–10 years, the target of iodine sufficiency has not been completely achieved [9,10,11,12,13,14,15].

Liguria is a small Italian region which extends along the coast of the Ligurian Sea (Riviera Ligure) and is bounded by mountains (Ligurian Appenines in the east and Maritime Alps in the west). The Provinces of Genoa and Savona are situated in the central part of the region, while those of La Spezia and Imperia lie in the east and west, respectively. The health services of Genoa and Savona also serve residents of the southern Piedmontese Provinces of Cuneo and Alessandria. Although Italy is considered a mildly iodine-deficient country, data from children in Liguria indicate a sufficient iodine intake [5,14]. However, we have very little direct information on the iodine intake of the adult population of Liguria. In 2018, in a cohort of 136 adults referred to our Center for Secondary Hypertension, we observed that, after salt restriction (by avoiding preserved foods), 24-h iodine excretion showed a non-significant decrease [16]. Indeed, 24-h iodine excretion lower than 100 µg was recorded in only 28 subjects (21%), suggesting an average iodine sufficiency in this cohort [16].

The aim of this study was to collect and examine the available data on urinary iodine concentration (UIC) and information on the use of iodized salt from all the clinical records of two endocrine outpatient clinics located in two provinces (Savona and Genoa) of the Liguria region in the period 2009–2018, after the nationwide salt iodization 55/2005 Law.

## 2. Materials and Methods

As part of an ongoing program of evaluation of dietary iodine intake in pediatric and adult populations at the University of Genoa, we searched a sample of medical files on adults living in Liguria and nearby districts (Imperia and south Piedmont) for data on the type of salt used and levels of UIC. No formal request to the Ligurian Ethics Committee was made, since on examination all patients provided written informed consent to the use of their clinical and biochemical data. The study was approved by the institutional review board.

We examined a total of 6025 clinical records from adult outpatients under evaluation for thyroid diseases in two endocrine clinics in the period 2010–2018. One clinic was a primary-level outpatient facility located in the Priamar Diagnostic Center in Savona, while the other was a secondary-level outpatient facility located in the Endocrine Unit at San Martino Polyclinic Hospital in Genoa. A subset of 486 clinical records in which iodine excretion had been reported were used for the present study. Patients were under evaluation for various thyroid diseases (nodular or autoimmune goiter, history of thyroid cancer, thyrotoxicosis, overt or subclinical hypothyroidism, destructive thyroiditis, etc.). Thyroidectomy was performed for goiter or cancer in 221 patients. The following information was collected: area of residence, body mass index (BMI; kg/m^2^), serum creatinine as an index of renal function, thyroid function test results (TSH, fT4), thyroid volume when reported, and daily dosages of levothyroxine (L-T4; µg/kg b.w.), when administered.

Data on age, gender, weight, height, disease history, type of salt consumed, use of medications, and thyroid testing performed near to the date of urine collection were sought in medical records. Thyroid volume was calculated as already reported, when data were available [17]. UIC was quantified in morning spot urine samples by means of commercial colorimetric methods. In the Savona district, a visual colorimetric method with laboratory assay *Celltech* (Alessandria, Italy) was used, while in the Genoa District a spectrophotometric method with *LTA* assay (Bussero, Milan, Italy) was used. Producers reported good reproducibility (*r* = 0.98) of methods. The sensitivity of the methods was 10 µg/L. Samples with UIC concentrations greater than 200 µg/L were re-evaluated after dilution. In accordance with the WHO (World Health Organization) standard, UIC values ≤ 20 µg/L indicate severe iodine deficiency; 20–49 µg/L moderate iodine deficiency; 50–99 µg/L mild iodine deficiency; 100–199 µg/L adequate status; 200–299 µg/L more than adequate status; and ≥ 300 µg/L iodine excess [18].

Data from patients on a low-iodine diet were not collected when this information was present in the clinical record. UIC values ≤ 20 µg/L or ≥ 300 µg/L were discarded from the subsequent statistical analysis because of the scant accuracy of the method in UIC values below 20 µg/L and above 300 µg/L. UIC values ≤ 20 µg/L or ≥ 300 µg/L were recorded in 45 (9.3%) and 26 (5.3%) patients, respectively. UIC values ≤ 20 µg/L were found in 32 patients from the Genova district, 12 from the Savona district, and one from another district. UIC values ≥ 300 µg/L were recorded in 13 patients from the Genova district and 13 from the Savona district.

The GraphPad 8.2 software (GraphPad, San Diego, CA, USA) was used for statistical analysis. To compare continuous data, we used the Kruskal–Wallis analysis of variance (ANOVA), followed by Dunn’s multiple comparison test, when applicable, and the Mann–Whitney test. Percentages were compared by means of Chi-square test. Correlations were evaluated by means of the Spearman test. Data are reported as mean ± standard deviation (SD). Significance was set at *p* < 0.05. Data collection and subsequent analysis were performed in compliance with the Helsinki Declaration.

## 3. Results

Some clinical data extracted from clinical records are reported in Table 1 and Table 2. Some significant differences in clinical diagnoses and thyroid drugs administered at the time of UIC evaluation were noted among the districts.

Most medical records did not report the type of salt consumed; data on salt use were found in 35%, 49%, and 33% of medical records from patients living in Genoa, Savona and other districts, respectively. These percentages were significantly different (*p* = 0.0007). Iodized salt use was reported only in 29%, 20%, and 13% of these patients, respectively.

The median UIC was 101.0 µg/L (25th–75th percentile 67.0–145.8 µg/L).

No difference (*p* = 0.13) was found between subjects with (*n* = 226, median 103.5 µg/L; 25th–75th percentile 69.2–150.8 µg/L) or without (*n* = 189, median 97.5 µg/L; 25th–75th percentile 66.2–138.0 µg/L) a thyroid gland.

UIC values from urinary spot samples collected in the years 2009–2013 (*n* = 143; median 105.0 µg/L, 25th–75th percentile 64.5–159.3 µg/L) and 2014–2018 (*n* = 272; median 97.5 µg/L, 25th–75th percentile 68.0–140.0 µg/L) were very similar (*p* = 0.32).

People living in Genoa showed slightly, not significantly (*p* = 0.17), lower UIC (*n* = 229; median 94.0 µg/L, 25th–75th percentile 63.3–141.0 µg/L) than those living in Savona (*n* = 162; median 105.0 µg/L; 25th–75th percentile 68.0–150.0 µg/L) and in the other nearby districts (*n* = 24; median 114.5 µg/L; 25th–75th percentile 80.0–199.8 µg/L).

**Figure 1:**
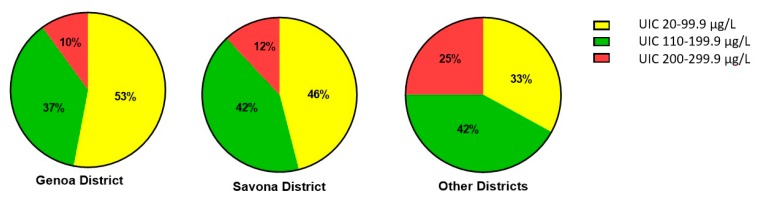
Percentage distribution of UIC (urinary iodine concentration) levels in the three districts evaluated.

Figure 1 reports the percentages of patients with UIC in the ranges 20–99.9 µg/L, 100–199.9 µg/L, 200–300 µg/L in the population samples evaluated. No significant difference in these percentages (*p* = 0.13) was observed among the districts, though a decreasing trend in moderate insufficiency was noted on moving from Genoa to Savona and the other districts.

No correlation emerged between UIC levels and patient age, BMI, creatinine, f-T4, TSH, thyroid volume, or L-T4 dosage (Table 3).

## 4. Discussion

The literature data show that UIC differs among populations worldwide. In New Zealand, a study involving 301 adults documented a median UIC that was 73 µg/L lower than the iodine intake estimated on the basis of food questionnaires (around 132 µg/day) [19]. A recent Chinese paper investigated the iodine status of 819 euthyroid adults from five Chinese cities, finding a median UIC of 134.0 µg/L and median creatinine-adjusted UIC ratio of 114.2 µg/g [20]. The authors of this paper also found variations in UIC in different age-groups and a tendency of TSH to increase with UIC, though neither correlation proved statistically significant [20]. In 2808 adults with a median UIC of 164.5 µg/L, Wang et al. [21] found a U-shaped relationship between iodine intake and thyroid autoimmunity, whereby a more than adequate iodine status (UIC 200–299.9 µg/L) seemed to be protective against thyroid autoimmunity [21]. They also documented younger age, lower BMI, and a lower prevalence of diabetes and hypertension in subjects with higher UIC [21]. An observational study involving 12,264 adult participants in the US National Health and Nutritional Examination Survey III (NHANES III, 1988–1994) documented a median UIC of 140 µg/L [22]. Over a follow-up of around 19 years, higher all-cause mortality was recorded in subjects with very high UIC (≥400 µg/L) than in those with normal UIC [22]. However, in our cohort, UIC values over 300 µg/L were mostly due to iodine contamination (e.g., amiodarone or iodized contrast media) rather than to excessive dietary intake, since the majority of these UIC values in clinical records corresponded to UIC assessed after a diagnostic procedure with iodinated contrast medium.

In our medical records, we found a median UIC of 101.0 µg/L, which is, according to the WHO standard [18], near to the adequate iodine status. However, median UIC appears insufficient if we consider only data collected in 2014–2018 (median UIC 97.5 µg/L), or only subjects living in Genoa (median UIC 94.0 µg/L) or those without a thyroid gland (median UIC 97.5 µg/L). This underlines the variability of iodine status in our region. We did not find correlations between UIC and age or BMI, as emerged from another, larger survey [21].

Owing to the method of our study, we probably do not have complete data on the use of iodized salt in Liguria. This information seems to be often undervalued during medical data collection. When this information was available, more than 70% of people stated that they did not use iodized salt. This percentage is worse than those previously documented in Italy. Indeed, Olivieri et al. [5] reported that iodized salt accounted for 55% of salt sales in Italy in 2012. Moreover, in recent surveys in northeastern Italy, 70–80% of families declared that they used iodized salt daily [9,11], while in a study conducted in Calabria the percentage of families using iodized salt was 64–82% in rural-urban areas [23]. Of the 136 subjects attending our Center for Secondary Hypertension, 62% declared using iodized salt, since, at the beginning of the study, all these patients had had a consultation with a dietitian and had received written nutritional advice [16].

Nutritional and cultural variables can influence iodine intake. Iodized salt and milk are the main foods that influence UIC in surveys conducted among Italian children [9,11]. Major barriers to iodine intake seem to be immigrant status and poverty, as documented in a cross-sectional survey recently conducted among around 3400 women referred to non-governmental organizations for their health needs [24]. However, the data we collected on both iodized salt and iodine excretion suggest insufficient attention to iodine intake in this cohort of subjects. More counseling on alimentary iodine intake in endocrine outpatients is therefore still needed.

Our paper has some limitations: (a) there were some differences between the two clinical settings: most of the patients from Genoa attended a secondary-level outpatient clinic for Thyroid Cancer, while most of those living in the other districts attended a primary-level facility; (b) relatively few records were available, as UIC is only sporadically assessed in clinical practice; (c) UIC was measured by two different laboratories using different commercial colorimetric methods, though good reproducibility was reported. Finally, we collected data on UIC in spot urine samples and not in 24-h samples, as spot urine samples are easy to obtain in an outpatient setting. In this regard, moreover, a 2013 WHO document on the use of UIC stated that morning or spot urine samples were adequate for the assessment of a population’s iodine status [18]. Indeed, this modality not only provides values for the assessment of iodine intake in school-age children, but is also applicable to adults, except pregnant and lactating women [19]. However, UIC determined on single random urine sample is inevitably affected by daily variations and variable urinary volume, and for this reason its accuracy is still debated [25,26].

## 5. Conclusions

In conclusion, this retrospective study showed a borderline status of iodine sufficiency in this cohort. We therefore suggest that endocrinologists should pay greater attention to nutritional behavior in order to improve iodine intake both in endocrine patients and in the general population.

## Figures and Tables

**Table 1:** Some clinical data (mean ± standard deviation; median, 25th and 75th percentiles) extracted from medical records of patients at the time of UIC (urinary iodine concentration) evaluation.

	Genoa District *n* = 229	Savona District *n* = 162	Other Districts (1) *n* = 24	Significance *p*
Age (years)	56.8 ± 15.2	56.2 ± 17.1	54.9 ± 14.5	0.83
Gender (female/male)	179/50	118/44	19/5	0.48
Clinical Diagnosis (%)				<0.0001 (a)
nodular goiter	15	38	17	
Graves’ disease	10	17	8	
Hashimoto’s thyroiditis	8	13	0	
thyroid cancer	51	18	58	
others	16	14	17	
Thyroid drugs (%)				<0.0001 (a)
levothyroxine	60	33	63	
methimazole	13	28	8	
none	27	39	29	
f-T4, free thyroxine (pmol/L)	16.3 ± 7.5 16.6; 12.7–20.1	14.3 ± 5.3 13.4; 10.6–17.0	16.2 ± 6.6 14.2; 12.5–20.9	0.0003 (b)
TSH, thyrotropin (mIU/L)	7.4 ± 19.4 1.0; 0.2–2.9	5.3 ± 16.3 1.2: 0.2–2.9	10.7 ± 26.1 1.1; 0.3–5.2	0.80
Thyroid peroxidase antibody (% of positivity)	45	44	37	0.92

(1) The other two provinces of the Liguria region (Imperia in the west and La Spezia in the east) and the southern provinces (Cuneo and Alessandria) of the Piedmont region. (**a**) Χ^2^ evaluated only between the Genoa and Savona districts; (**b**) Dunn’s multiple comparison test: Genoa district vs. Savona district *p* = 0.002.

**Table 2:** Some other clinical data extracted from medical records of patients at the time of UIC evaluation.

	Mean Value	SD	Min.	Max.	Median Value
Age (*n* = 486) (years)	56.57	15.84	18	86	58
Weight (*n* = 473) (kg)	69.29	14.44	37	117	66
Height (*n* = 455) (m)	163.12	9.30	130	196	162
BMI (*n* = 455) (kg/m^2^)	26.13	5.13	16.7	48.7	25.5
BSA (*n* = 455) (m^2^)	1.76	0.21	1.19	2.44	1.72
Creatinine (*n* = 339) (mg/dL)	0.84	0.23	0.4	2.3	0.8
TSH (*n* = 479) (mU/L)	7.57	21.16	0.01	163.3	1.06
fT4 (*n* = 463) (ng/L)	12.12	5.38	0.2	44.1	11.7
Thyroid volume (*n* = 188) (mL)	30.66	124.61	1	1705	13.55
L-T4 (*n* = 231) (µg/kg die)	1.34	0.42	0.19	2.59	1.34

**Table 3:** Correlations between UIC levels and other clinical and hormonal parameters evaluated.

Variable	Pair *n*	Correlation rS	Significance *p*
Age (years)	415	−0.01	0.77
BMI (kg/m^2^)	380	0.08	0.09
Creatinine (µmol/L)	289	−0.01	0.80
f-T4 (pmol/L)	393	−0.01	0.92
TSH (mIU/L)	405	−0.04	0.36
Thyroid volume (mL)	166	0.08	0.39
L-T4 (µg/kg b.w./day)	187	−0.03	0.65
